# Targeting the Microtubule-Network Rescues CTL Killing Efficiency in Dense 3D Matrices

**DOI:** 10.3389/fimmu.2021.729820

**Published:** 2021-08-17

**Authors:** Renping Zhao, Xiangda Zhou, Essak S. Khan, Dalia Alansary, Kim S. Friedmann, Wenjuan Yang, Eva C. Schwarz, Aránzazu del Campo, Markus Hoth, Bin Qu

**Affiliations:** ^1^Biophysics, Center for Integrative Physiology and Molecular Medicine (CIPMM), School of Medicine, Saarland University, Homburg, Germany; ^2^INM-Leibniz Institute for New Materials, Saarbrücken, Germany; ^3^Molecular Biophysics, CIPMM, School of Medicine, Saarland University, Homburg, Germany

**Keywords:** CTLs, collagen, dense matrices, microtubules, migration, nuclear deformation, 3D killing

## Abstract

Efficacy of cytotoxic T lymphocyte (CTL)-based immunotherapy is still unsatisfactory against solid tumors, which are frequently characterized by condensed extracellular matrix. Here, using a unique 3D killing assay, we identify that the killing efficiency of primary human CTLs is substantially impaired in dense collagen matrices. Although the expression of cytotoxic proteins in CTLs remained intact in dense collagen, CTL motility was largely compromised. Using light-sheet microscopy, we found that persistence and velocity of CTL migration was influenced by the stiffness and porosity of the 3D matrix. Notably, 3D CTL velocity was strongly correlated with their nuclear deformability, which was enhanced by disruption of the microtubule network especially in dense matrices. Concomitantly, CTL migration, search efficiency, and killing efficiency in dense collagen were significantly increased in microtubule-perturbed CTLs. In addition, the chemotherapeutically used microtubule inhibitor vinblastine drastically enhanced CTL killing efficiency in dense collagen. Together, our findings suggest targeting the microtubule network as a promising strategy to enhance efficacy of CTL-based immunotherapy against solid tumors, especially stiff solid tumors.

## Introduction

Cytotoxic T lymphocytes (CTLs), which are activated CD8^+^ T cells, compose an essential arm of the immune system to fight aberrant cells like tumorigenic and pathogen-infected cells (1, 2). CTLs recognize their targets *via* engagement of T cell receptors (TCRs) with the cognate antigens presented on the surface of target cells (3–5). Once the matching antigens are identified, activation of TCRs triggers the downstream signaling cascades to re-orientate the CTL killing machinery towards the contact site, termed immunological synapse (IS) (6–8). The major killing mechanisms employed by CTLs are lytic granules (LGs) and Fas/FasL pathway (9). LGs contain cytotoxic proteins such as pore-forming protein perforin and serine-protease granzymes (10). Upon target recognition, LGs are enriched at the IS and are eventually released into the synaptic cleft to induce destruction of target cells (11, 12). In order to locate their targets, CTLs need to migrate through peripheral tissues across the three-dimensional (3D) extracellular matrix (ECM).

The ECM is a 3D network of fibrous structural proteins, collagen being the major constituent (13). The ECM of solid tumors is often densified, creating a physical hindrance that impairs infiltration of CTLs, accompanied with diminished killing efficiency (14, 15). Proliferation of T cells is also reduced in a high-density matrix (16). Using an elegant 2.5 D engineered platforms with grooves, a recent study shows that the dynamics of microtubule plays an important role in migration of primary human CD4^+^ T cells, and this tendency is verified with mouse CD8^+^ T cells (17). However, the impact of dense ECM on killing efficiency of CTLs *per se* still remains largely uncharacterized.

The cytoskeleton, including actin and the microtubule network, plays a pivotal role in regulating migration of CD8^+^ T cells (18, 19). During migration, T cells generate protrusions at the leading edge, which are mainly driven by polymerization of actin (20). The myosin-mediated contraction of F-actin generates force to retract the uropod, the rear part of the cell, and enables T cells to move forward (21). In T cells, myosin IIA is the predominantly expressed member of the myosin family (20). Blockage of myosin IIA activity results in deficiency of uropod retraction and therefore significantly impairs T cell migration (21). During T cell migration, the microtubule-organizing center (MTOC) is located at the uropod (22). Abrogation of microtubule polymerization does not hinder T cell migration (23).

Nuclear deformability serves as a rate limiting factor of cell migration through physical restricted 3D spaces (24, 25). To pass the restricted space between pillars, or narrow channel, the nucleus has to be deformed by force from cytoskeleton to fit the size of space (24, 26, 27), which also facilitates cell migration along the path of least resistance in a complex environment (25). Further reports showed that the nucleus shape and its shape changes are correlated with the velocity of cell migration (28, 29).

In this work, we investigated CTL-mediated cytotoxicity against tumor cells in collagen matrices of different densities that mimic physiological and pathological microenvironments. We confirmed that CTL killing efficiency was substantially reduced in dense collagen matrices. Although the killing machinery *per se* remained intact, migration of CTLs was significantly impaired. Migrating CTLs in dense matrices exhibit deformed nuclei, the extent of which correlated with migration velocity, indicating that flexibility of CTL nuclei is pivotal to CTL migration in 3D. We found that nuclei flexibility is regulated by the microtubule networks. Importantly, disruption of microtubule but not actin polymerization can rescue the impaired migration as well as the reduced cytotoxic efficiency of CTLs in dense collagen matrices.

## Materials and Methods

### Antibodies and Reagents

All chemicals not specifically mentioned are from Sigma-Aldrich (highest grade). All inhibitors not specifically mentioned are from Cayman Chemical. The following antibodies were used: Alexa Fluor 647 anti-human CD3 antibody (UCHT1, BioLegend), Alexa Fluor 488 anti-human Granzyme A antibody (CB9, BioLegend), Alexa Fluor 647 or Brilliant Violet (BV) 510 anti-human perforin antibody (dG9, BioLegend), Alexa Fluor 647 anti-human granzyme B antibody (GB11, BioLegend), Brilliant Violet (BV) 421 anti-human CD178 (Fas-L), anti α-Tubulin mAb antibody (DM1A, Cell Signaling Technology), and Alexa Fluor 405 conjugated goat anti-mouse IgG (H+L) cross-absorbed secondary antibody (ThermoFisher Scientific). All isotype controls of fluorescence conjugated primary antibodies are from BioLegend. The following reagents were used: Hoechst 33342 (ThermoFisher Scientific), Alexa Fluor 488 or Alexa Fluor 568 phalloidin (ThermoFisher Scientific), Atto 488 NHS ester ((ThermoFisher Scientific), collagenase type I (ThermoFisher Scientific), FibriCol^®^ type I collagen Solution (Bovine, Advanced Biomatrix).

### DNA Constructs

For pGK-puro-pCasper-pMax (referred to as pCasper-pMax in the manuscript), the vector backbone used for generation of this plasmid is a kind gift from Ulrich Wissenbach (Saarland University) who previously modified the AMAXA vector (Lonza) by replacing the sequence encoding GFP with a linker sequence encoding a multiple cloning site (pMAX). In a first step, the sequence encoding pCasper was amplified from pCasper3-GR (evrogen #FP971) with the following primers introducing an XhoI recognition site at both ends of the amplicon. Forward primer: 5’-CTCGAGGCCACCATGGTGAGCGAG -3’, reverse primer: rev 5’-GACGAGCTGTACCGCTGACTCGAG-3’. The amplicon was subcloned into the XhoI site of pMAX. In a second step, the sequence encoding puromycin resistance was introduced into the intermediate plasmid under the control of 3-phosphoglycerate kinase promoter (PGK-1). The PGK-1-Puromycin sequence was amplified out of pGK-Puro-MO70 vector backbone (Alansary et al., BBA 2015) using the following primers introducing SacI recognition sites at both ends of the amplicon to insert it into the Sac-I site of the intermediate plasmid. Forward primer: 5’-GAGCTCAATTCTACCGGGTAGGGGA-3’, reverse primer 5’-GCAAGCCCGGTGCCTGAGAGCTC-3’. The final plasmid is named pGK-puro-pCasper-pMAX. Final and intermediate plasmids were controlled by endonuclease digestion patterns and sequencing. As a gift from William Bement (Addgene plasmid # 26741). Histone 2B-GFP was a gift from Geoff Wahl (Addgene plasmid # 11680). LifeAct-mRuby was a kind gift from Roland Wedlich-Söldner (University of Muenster). pmEGFP_a_tubulin_C1 was a gift from Daniel Gerlich (Addgene plasmid # 21039). pmCherry-C1 mCherry-NLS was a gift from Dyche Mullins (Addgene plasmid # 58476).

### CTL Preparation, Cell Culture, and Nucleofection

Peripheral blood mononuclear cells (PBMCs) were obtained from healthy donors as described before (30). Briefly, leukocyte reduction chambers were flushed with Hank’s Balanced Salt Solution and loaded on a standard density gradient Leukocyte separation medium (LSM 1077, PAA). PBMCs were isolated by a density gradient centrifugation (450 g, 30 min), and remaining red blood cells were removed by the lysis buffer (155 mM NH_4_Cl, 10 mM KHCO_3_, 0.1 mM EDTA, pH=7.3). Human primary CD8^+^ T Cells were negatively isolated from PBMCs using Dynabeads™ Untouched™ Human CD8 T Cells Kit (ThermoFisher Scientific) or Human CD8^+^ T Cell Isolation Kit (Miltenyi Biotec), stimulated with Dynabeads™ Human T-Activator CD3/CD28 (ThermoFisher Scientific) with 17 ng/ml of recombinant human IL-2 (ThermoFisher Scientific). MART-1-specific CD8^+^ T-cell clones were generated by Friedmann et al. (31). All CD8^+^ T cells were cultured in AIM V medium (ThermoFisher Scientific) containing 10% fetal calf serum (FCS) and 1% Penicillin-Streptomycin. For nucleofection, CD3/CD28 beads were removed 48 hours after stimulation and 5 × 10^6^ CTLs were electroporated with 2 μg plasmid using 4D-Nucleofector (Lonza). Medium was changed 6 hours after nucleofection and transfected cells were used 24-36 hours after electroporation.

Raji and NALM-6 cells were cultured in RPMI-1640 medium (ThermoFisher Scientific) containing 10% FCS and 1% Penicillin-Streptomycin. NALM-6 pCasper cells were generated by Knörck et al. (32) and were cultured in RPMI-1640 in the presence of puromycin (0.2 µg/ml). SK-Mel-5 cells were transfected with pCasper-pMax using jetOPTIMUS^®^ DNA Transfection Reagent (Polyplus-transfection) following the manufacturer’s instructions and then cultured in MEM medium (ThermoFisher Scientific) containing 10% FCS and 1% penicillin-streptomycin. All cells were cultured at 37°C with 5% CO_2_.

### Preparation of Collagen Matrix and Cell Embedding

Collagen hydrogels were prepared following previous protocols (33). Briefly, bovine collagen type I stock solution (10 mg/ml) was neutralized with 0.1 N NaOH solution on ice to reach pH 7.0-7.4. 10×PBS was added into the neutralized collagen to a dilution factor of 1:10. The collagen solution was further diluted with PBS to the final concentrations. Cells were resuspended in the collagen solution and the mixture was left for 1 hour at 37°C with 5% CO_2_ (if not mentioned otherwise) for fibrillation in 96-well plates. In order to increase the stiffness of the collagen matrix, the collagen stock solution was first diluted to a concentration of 3 mg/ml with 0.1% acetic acid with 100 mM ribose (34). These collagen solutions were kept at 4°C for 5 days, and then used for cell encapsulation as described before.

### Killing Assay in 3D With the High-Content Imaging Setup

For killing assays, we used either NALM-6 cells stably expressing apoptosis reporter pCasper-pMax or SK-Mel-5 cells transiently transfected with pCasper-pMax (referred to as NALM-6-pCasper or SK-Mel-5-pCasper, respectively) as target cells. NALM-6-pCasper were pulsed with staphylococcal enterotoxin A (SEA, 0.1 µg/ml) and SEB (0.1 µg/ml) at 37°C with 5% CO_2_ for 40 min prior to killing assays. Target cells were resuspended in chilled collagen solution, and transferred in 96-well plates. After centrifugation at 4°C (200 g, 7.5 min), collagen was solidified in the incubator for 1 hour. CTLs were then added from the top if not mentioned otherwise. For the inhibitor-treatment, CTLs were added on top of solidified collagen in medium containing the corresponding inhibitor or vehicle. The effector to target (E:T) ratio for pre-mixed condition is 1:1. For the condition that CTLs were added on top of the collagen, the E:T ratio is 5:1 or 1:1 for CTL : NALM-6-pCasper and MART1 specific CTLs: SK-Mel-5-pCasper, respectively.

Images were acquired by ImageXpress (Molecular Devices) with Spectra X LED illumination (Lumencor) at 37°C with 5% CO_2_ for 12 to 24 hours. As described previously (35), fluorescence of pCasper-pMax was acquired using LEDs 470/24 for excitation and the following filter sets (Semrock): Ex 472/30 nm, Em 520/35 nm for GFP and Em 641/75 nm for RFP/FRET. A 20× S Fluor 0.75 numerical aperture objective (Nikon) was used. The killing efficiency was calculated as (1-N_exp_(t)/(N_exp_(t_0_)×N_live_(t)/N_live_(t_0_))×100%.

(N_live_: number of live target cells in the control wells without CTLs; N_exp_: number of live target cells in the experimental wells; t_0_: the first time point of the measurement; t: end time point of the measurement).

### Immunostaining and Flow Cytometry

CTLs were fixed with pre-chilled 4% paraformaldehyde (PFA) after recovery from collagen degraded with collagenase. Then cells were washed twice with PBS/0.5% BSA, permeabilized and blocked with 0.1% saponin in PBS containing 5% FCS and 0.5% BSA, and then stained with the indicated primary antibody or Alexa Fluor 488 Phalloidin for 30 min at room temperature followed by staining of Alexa Fluor 405 labeled secondary antibody if the primary antibody was not fluorophore-conjugated. Flow cytometry data were acquired using a FACSVerse™ flow cytometer (BD Biosciences) and were analyzed with FlowJo v10 (FLOWJO, LLC).

### 3D Live-Cell Imaging Using Light-Sheet Microscopy

As described previously (33), collagen with 10 × 10^6^ CTLs/ml polymerized in the capillary at 37°C with 5% CO_2_ for 2 hours. Afterwards, the samples were scanned with light-sheet microscopy Z1 (Zeiss) at 37°C for 30 min with an interval of 30 sec and a z-step size of 1 µm. A 20× objective (W Plan-Apochromat, N.A. 1.0) was used. Excitation was realized by two lasers, 488 and 561 nm. Emission was filtered *via* Em525/40nm and Em 585 LP filters. The images were acquired with ZEN 2014 SP1 Hotfix 2 software. Trajectories of CTLs and nuclear irregularity index (NII) were determined and analyzed with Imaris 8.1.2 (containing Imaris, ImarisTrack, ImarisMeasurementPro, ImarisVantage from Bitplane AG). The nuclei or the cell bodies were detected automatically by ImageJ/Fiji based on the corresponding fluorescence, and parameters (circularity and Feret’s diameter) were analyzed with ImageJ/Fiji. Nucleus deformability is the average of nucleus sphericity change between two neighbor time points.

### Visualization of CTL Migration in a Planar 3D Collagen Matrix With Zeiss Observer Z.1

CTLs (5 × 10^6^ cells/ml) were resuspended in collagen solution with or without Calcein labeled target cells (5 × 10^6^ cells/ml). Cell/collagen mixture (3 µl) was pipetted as a droplet onto the center of an Ibidi μ-dish (Ibidi GmbH). Then a Sigmacote^®^ (Merck) coated glass coverslip (5 mm, Orsatec GmbH) was carefully placed on top to flatten the droplet (calculated thickness around 150 µm). The Ibidi μ-dish was closed with the lid and incubated at 37°C with 5% CO_2_ for 1 hour. After collagen polymerization, the glass coverslip was removed from collagen matrix. For migration assay, CTLs were either non-fluorescent (in presence with target cells) or stained with 1 µg/ml Hoechst 33342 in AIMV (10% FCS) medium for 30 min (without target cells), and then were incubated in fresh AIMV (10% FCS) medium for another 30 min in presence of inhibitors or vehicles as indicated in the figure legends. Raji cells and unlabeled CTLs were mixed. The images were acquired with Zeiss Observer Z.1 for 30 min or 3 hours at 37°C with 5% CO_2_ with a Zeiss Colibri LED illumination system and 20× objectives (Fluar 20×/0.75 M27 Air). Images were taken using an AxioCamM1 CCD camera and AxioVision 4.1.8. The images of cell migration and nuclear irregularity index (NII, measured as nuclear circularity) were analyzed using Imaris 8.1.2. Migration trajectories in presence of target cells were analyzed by Fiji. Nuclear deformationability is defined as standard deviation of NII. To quantify CTL search efficiency, CTLs were randomly selected from the CTLs that were observed for the whole period. The probability for CTLs finding at least one target within 3 hours was quantified. From these CTLs, the time of CTL contacting the first target cells was quantified, if this CTL could find at least one target cell within 3 hours. Nucleus deformability is the average of nucleus circularity change between two neighbor time points.

### Confocal Microscopy and Determination of Nuclear Deformation

For the fixed sample, CTLs were fixed at indicated time points with 4% PFA and permeabilized with 0.3% Triton-100 with 5% FCS in PBS, followed by staining with indicated antibodies or fluorescent dyes according to the manufacturers’ instructions. Images were acquired by confocal microscopy LSM 710 with a 63× objective (N.A. 1.4) and a Nikon E600 camera using ZEN software. The nuclear irregularity index (NII, measured as nuclear circularity from maximum intensity projection) was analyzed with ImageJ. The distance between nucleus and MTOC was analyzed with Imaris 9.6.

### Visualization of Collagen Structure and Determination of Porosity

After collagen polymerization in the capillary, collagen was stained with Atto 488 NHS ester in PBS (50 µM) at room temperature for 15 min. Afterwards, the collagen matrix was washed by PBS twice. Matrix structure of collagen was visualized by light-sheet microscopy with a 20× objective (W Plan-Apochromat, N.A. 1.0). Collagen pore size was measured in the middle slice of the z-stack by Fiji (BIOP version) with Max Inscribed Circles plugin as described elsewhere (36).

### Shear Rheology Stiffness Measurement

Rheology measurements with different concentrations of bovine collagen were performed using DHRIII Rheometer (TA Instruments). 50 µl of the neutralized collagen solution (pH 7.0-7.4) was placed between two parallel plates of 12 mm diameter pre-heated to either 25°C or 37°C. The shear moduli were measured at frequency ω – 3 rad/s at 37°C as described previously (37). All experiments were performed in triplicates.

### Viability Assay

CTLs were embedded in 5 mg/ml collagen in a 96-well plate. After collagen polymerization, 100 µl AIMV medium with 10% FCS, 1 µg/ml propidium iodide, and nocodazole or DMSO was added. Images were acquired by ImageXpress with Spectra X LED illumination (Lumencor) using LEDs 542/27 for excitation. The filter set was Ex 542/27 nm and Em 641/75 nm. A 20× S Fluor 0.75 numerical aperture objective (Nikon) was used. The images were acquired at 37°C with 5% CO_2_ every 1 h for 12 h.

### Ethical Considerations

Research carried out for this study with healthy donor material (leukocyte reduction system chambers from human blood donors) is authorized by the local ethic committee [declaration from 16.4.2015 (84/15; Prof. Dr. Rettig-Stürmer)].

### Statistical Analysis

Data are presented as mean ± SD. GraphPad Prism 6 Software (San Diego, CA, USA) was used for statistical analysis. If the number of data points is smaller than 8, the differences between two columns were analyzed by the Student’s t-test. Otherwise, the data were first examined for Gaussian distribution. If the dataset fit Gaussian distribution, the differences between two columns ware analyzed with the Student’s t-test, otherwise with the Mann-Whitney test.

## Results

### CTL Killing Efficiency and Motility Is Substantially Impaired in Dense Collagen

**Figure 1:**
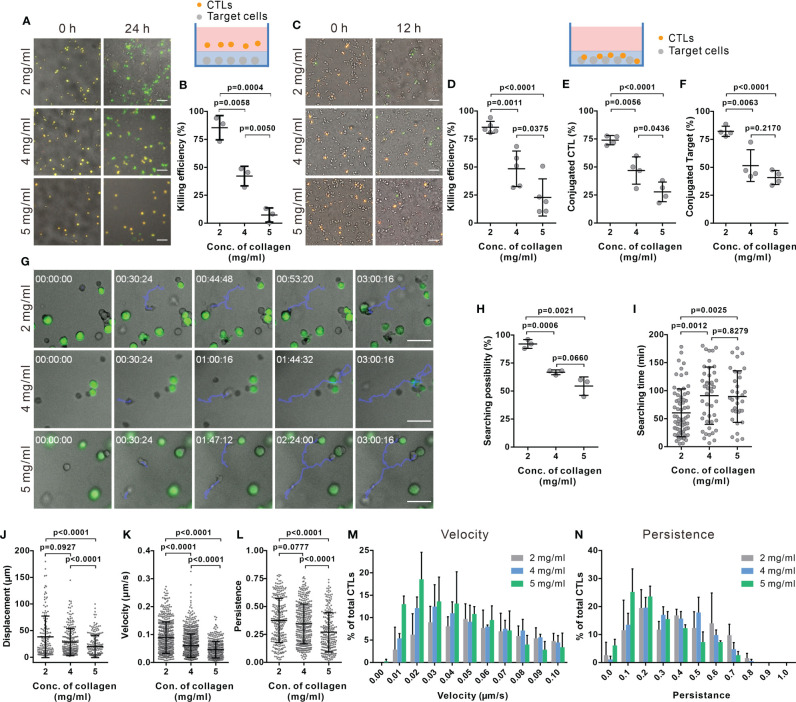
The killing efficiency of CTLs is substantially decreased in dense collagen matrices. **(A, B)** CTL killing efficiency is impaired in dense collagen matrices. Target cells (SEA/SEB pulsed NALM-6-pCasper) were embedded in collagen in absence (**A, B,** 3 donors, E (effector cells): T (Target cells) = 5:1) or presence of CTLs (**C, D,** 5 donors, E:T=1:1). Images were acquired using ImageXpress with Spectra X LED illumination (Lumencor) for 24 hours **(A, B)** or 12 hours **(C, D)**. Orange and green color indicates live and apoptotic target cells, respectively. **(E, F)** Fraction of CTLs in conjugation with target cells **(E)** or target cells in conjugation with CTLs **(F)** was analyzed at 1 hour after polymerization (4 donors). **(G–I)** Searching efficiency of CTLs is reduced in dense collagen. Target cells (SEA/SEB pulsed Raji) were loaded with calcein (green) and embedded in planar collagen with CTLs. Migration was visualized using Cell Observer (20× objective) at 37°C for 3 hours. Representative tracks are shown in **(G)** (blue lines). The likelihood to find a target within 3 hours is shown in **(H)** (3 donors). Time required for CTLs to find their first target (if the cell found at least one target cell) is shown in **(I)**. **(J–N)** Characterization of CTL migration in collagen matrices. CTLs were transfected with histone 2B-GFP and LifeAct-mRuby, and then embedded in 3D collagen. Migration was visualized using light-sheet microscopy (20× objective) every 30 sec for 30 min. The nuclei positions were identified with Imaris to analyze displacement **(J)**, velocity **(K)**, and persistence of CTLs **(L)** (3 donors). The distribution of CTL velocity and persistence is shown in **(M, N)**, respectively. One dot represents either one donor (in **B, D–F, H**) or one cell (in **I–L**). Results are presented as Mean ± SD. The unpaired Student’s t-test was used for statistical significance, except **(I–L)** (the Mann-Whitney test was used). Scale bars are 50 μm.

To investigate the impact of collagen density on the killing efficiency of CTLs, we used collagen matrices prepared at three different collagen concentrations (2, 4, and 5 mg/ml). Primary human CD8^+^ T cells were stimulated with anti-CD3/anti-CD28 antibody-coated beads to obtain CTLs. Target cells (NALM-6-pCasper) were embedded in the collagen matrices and CTLs were settled on top of the gels, as depicted in **Figure 1A** (schematic diagram). Target cells stably expressing apoptosis reporter pCasper-pMax, a GFP-RFP FRET pair linked by a sequence containing caspase recognition site (DEVD), allowing the detection of cell death (32). Apoptotic target cells switches the fluorescence to green as the linker between the GFP-RFP FRET pair is cleaved and necrotic target cells show a complete loss of fluorescence (35) (**Supplementary Figure 1A**). Using a high-content imaging setup (ImageXpress), we observed that 85.4 ± 10.9% of target cells in 2 mg/ml collagen were killed by either apoptosis or necrosis after 24 hours. The fraction of apoptotic and necrotic target cells eliminated by CTLs dropped to 42.1 ± 8.8% and 7.3 ± 6.3% in collagen matrices in 4 and 5 mg/ml collagen matrices, respectively. (**Figures 1A, B**). The same trend was observed at earlier time points (12 hours, **Supplementary Figure 1B**).

To exclude a possible influence of the CTL infiltration step from the gel surface into the gel interior, similar experiments were performed by embedding the CTLs with the target cells in the collagen gel. The cytotoxic efficiency of CTLs was similar to the experiment that included infiltration (**Figures 1A–D**). These results indicate that CTL killing efficiency was solely diminished by the dense collagen network.

To unravel the underlying mechanisms for a reduced CTL killing efficiency in dense collagen matrices, we first examined lytic granule and FAS/FASL pathways. To test the expression of cytotoxic proteins, we recovered CTLs from collagen matrices using collagenase. Control experiments showed that the collagenase treatment does not alter the protein level on T cells, e.g. CD3 expression (**Supplementary Figure 2A**). No significant differences in the expression of cytotoxic proteins (perforin, granzyme A and granzyme B) were observed in CTLs extracted from the collagen hydrogels of the three different concentrations (**Supplementary Figure 2B**). FasL expression was also examined, which was at very low levels for all three concentrations (**Supplementary Figure 2C**). Interestingly, in dense collagen, the fraction of CTLs conjugated with targets (**Figure 1E**) and the fraction of target cells conjugated with CTLs (**Figure 1F**) were significantly reduced. These results indicate that the impaired CTL killing efficiency in dense collagen is not owed to changes in the main components of the killing machinery but rather to a reduced search efficiency.

We next investigated the search efficiency of CTLs in collagen in detail using live-cell imaging. **Figure 1G** shows that a migrating CTL (highlighted by the blue track) in a 2 mg/ml collagen needs around 45 min to find the first target, whereas CTLs in 4 and 5 mg/ml ECM need about 100 min (lower panels of **Figure 1G**). In 2 mg/ml collagen matrices, most CTLs (92.0 ± 4.0%) found at least one target cell within 3 hours; whereas this likelihood was reduced to 66.8 ± 2.1% or 54.4 ± 8.2% in 4 and 5 mg/ml matrices (**Figure 1H**). Analyzing only the CTLs that found target cells, in 4 and 5 mg/ml collagen matrices, these needed about 90 min to find the first target but only about 60 min in 2 mg/ml collagen (**Figure 1I**). These results suggest that the probability of CTLs to find their targets is decreased in dense collagen matrices.

The mobility of CTLs in the matrix is a key factor for optimal search efficiency. To quantify motility, we analyzed displacement, migration velocity, and persistence (indicating how directed the migration is) of CTLs inside the collagen matrices using light-sheet microscopy. In dense collagen matrices (4 and 5 mg/ml), the displacement of CTLs (distance between the starting point and the end point) was reduced (**Figure 1J**, **Supplementary Figure 3, Supplementary Movies 1–3**). Analysis of trajectories in 3D collagen shows that velocity and persistence of migrating CTLs decreased with increasing collagen concentration (**Figures 1K, L**, **Supplementary Figure 3**). It was also observed that CTL migration was impaired in the high concentration of collagen when target cells are present (**Supplementary Figures 4A–C**). Analysis of velocity and persistence distributions reveals a higher fraction of CTLs with low velocity and low persistence in dense collagen (5 mg/ml, **Figures 1M, N**). Moreover, migration velocity is negatively correlated with the time of CTL searching target cells (**Supplementary Figure 4D**). In summary, our findings suggest that in a dense matrix, CTL migration is hindered, and this is likely the reason for longer search time and reduced cytotoxic efficiency.

### CTL Migration in 3D Collagen Matrices Is Regulated by the Matrix Stiffness and Porosity

**Figure 2:**
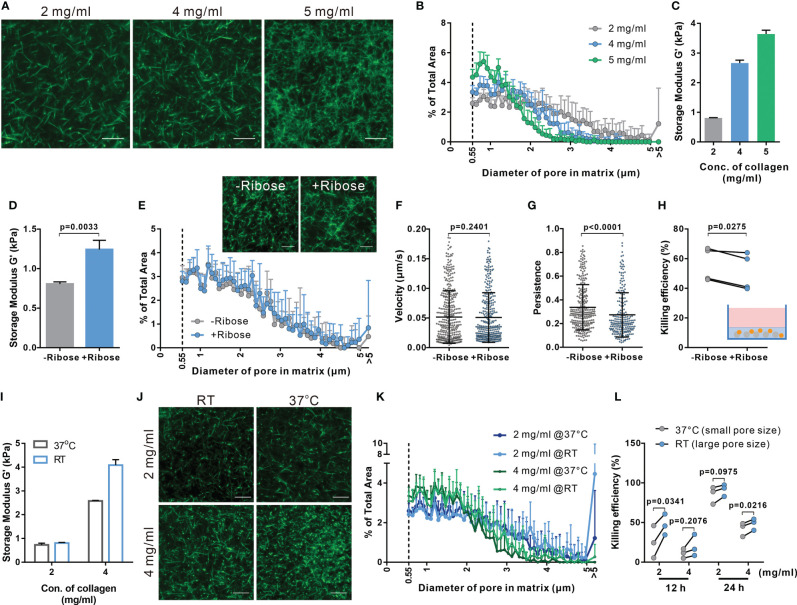
Both enhanced stiffness and reduced pore size contribute to impaired CTL killing efficiency in dense matrices. **(A, B)** Characterization of porosity in collagen matrices with different densities. Structure of collagen stained with Atto 488 NHS ester was visualized using light-sheet microscopy with a 20× objective **(A)**. The distribution of pore size in these matrices is shown in **(B)**. **(C)** Stiffness of collagen matrices was determined by the storage modulus using rheometer. **(D–H)** Adding ribose increases stiffness of collagen (2 mg/ml) without affecting the pore size. Stiffness and pore size of the respective conditions is shown in **(D, E)**, respectively. **(F–H)** Stiffening collagen leads to reduced CTL killing efficiency in 3D. Hoechst 33342-stained CTLs were embedded in a planar 3D collagen matrix. Migration was visualized using Cell Observer (20× objective) for 30 min **(F, G)**. Migration velocity and persistence are shown in **(F, G)**, respectively. To determine killing efficiency, CTLs were embedded with NALM-6-pCasper (E:T=1:1) for 4 hours **(H)**. **(I–K)** Pore size is increased by polymerizing collagen at RT. Stiffness of collagen matrices was determined by the storage modulus using rheometer **(I)**. The structure was visualized using light-sheet microscopy (20× objective) **(J)**. The pore size at each condition is shown in **(K)**. **(L)** Enlarged pore size improves CTL killing in 3D. NALM-6-pCasper cells were embedded in the matrices and CTLs were added from the top (E:T=5:1). Results are presented as Mean ± SD from 2 **(A, B, E, J, K)** or 3 independent experiments (for **F–H**, **L**, results are from 3 donors). One dot stands for either one cell (in **F**, **G**) or one donor (in **H**, **L**). For statistical significance, the unpaired Student’s t-test **(D)**, the Mann-Whitney test **(F, G)** or the paired Student’s t-test **(H, K)** was used. Scale bars are 10 μm.

We examined the correlation between CTL killing efficiency and the physical properties of collagen matrix, including pore size and the stiffness. We fluorescently labeled collagen to visualize its structure (**Figure 2A**) to determine pore size (**Supplementary Figure 5**) and found the pore size was decreased with increasing density (**Figures 2A, B**). Collagen stiffness was determined with the storage modulus G’ measured by rheology, which increased from 0.81 kPa to 3.64 kPa with collagen density between 2 and 5 mg/ml (**Figure 2C**). To study which of these two features contributes to the impaired CTL migration in collagen matrix, we modified the collagen matrix. To widen the range of stiffness keeping the pore size constant, we also prepared collagen matrices with 100 mM ribose (34), which showed increased storage modulus up to 1.24 kPa (**Figure 2D**), without affecting the pore size (**Figure 2E**). We observed no changes in CTL velocity with increasing stiffness (**Figure 2F**), while the persistence was reduced (**Figure 2G**). In line with this result, the cytotoxic efficiency of CTLs was reduced in collagen matrices prepared in the presence of ribose (**Figure 2H**). These results indicate that stiffness is involved in regulating CTL migration persistence, which could influence CTL killing efficiency.

To increase the pore size of the matrix maintaining a constant collagen concentration, the fibrillation step was performed at room temperature [instead of 37°C (38)]. Noticeably, collagen matrices obtained at 2 mg/ml collagen concentration showed similar shear moduli, while 4 mg/mL matrices with larger pores showed an increased stiffness (**Figure 2I**). This could be attributed to slower fibrillation kinetics of collagen at RT (39, 40). Nevertheless, in matrices with larger pore sizes (**Figures 2J, K**) the cytotoxic efficiency of CTLs was enhanced (**Figure 2L**). Together, these results suggest that smaller pore size and higher stiffness in dense collagen matrices lead to impaired CTL killing efficiency as a result of hindered migration, whereby CTL migration persistence is mainly determined by matrix stiffness while the velocity is likely determined by the pore size of the matrix.

### Deformability of Nucleus Is a Limiting Factor for CTL Migration in Dense ECM

**Figure 3:**
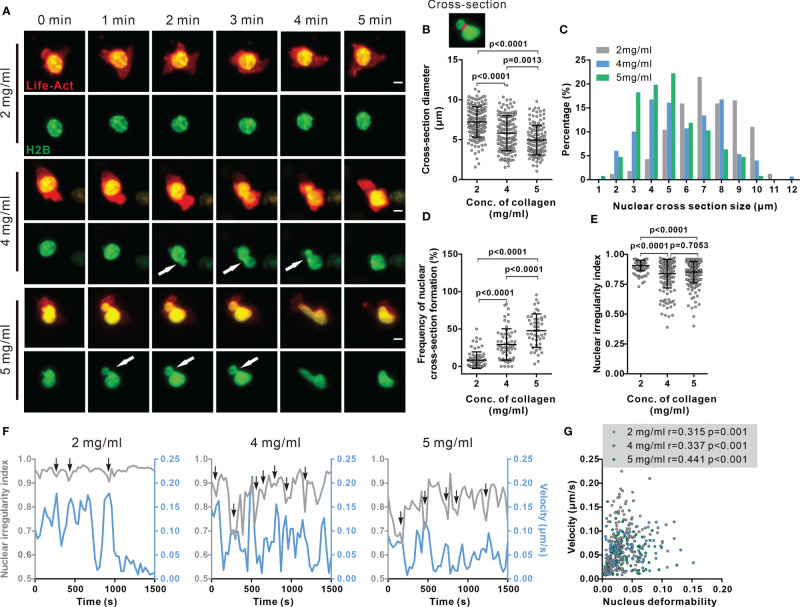
Nuclear deformation is correlated with migration velocity in dense collagen matrices. **(A–E)** Nuclear deformation in CTLs is enhanced upon the increase in matrix density. CTLs transfected with Histone 2B-GFP (green) and LifeAct-mRuby (red) were embedded in collagen. Migration was visualized using light-sheet microscopy (20× objective) at 37°C every 30 sec for 30 min. Exemplary cells are shown in **(A)**. Cross-sections are pointed by the arrowheads. Scale bars are 5 µm. Quantification of cross-section diameter, their distribution and duration is shown in **(B)** (indicated as red-line in the inset), **(C**, **D)**. The nuclear irregularity index of the cells in **(A–D)** is shown in **(E)**. Results are presented as Mean ± SD for **(B**, **D**, **E)**. All results are from 3 donors. The Mann-Whitney test was used for statistical significance. **(F**, **G)** Migration velocity of CTLs is positively correlated with nuclear deformation. NII (sphericity) and velocity as function of time in exemplary cells are shown in **(F)**. Correlation between velocity and nuclear deformability is shown in **(G)**. One dot represents one cell. Correlation coefficient r is analyzed with Spearman’s correlation. All results are from 3 donors.

As the stiffest organelle in cells, the nucleus is essential for decision making of migration direction for immune cells migrating through restricted space (24, 26, 27). Therefore, we examined the morphology of the nucleus in impaired CTL migration in dense matrices. First, we noticed that the nuclear morphology was deformed to an hour-glass shape in migrating CTLs (**Figure 3A**, **Supplementary Movies 4–6**). To quantify the extent of this nuclear deformation, we analyzed the diameter of cross-sections (the shortest intersection of the hour-glass shape), as well as the sphericity (how closely an object resembles a sphere). The cross-section diameter was decreased in matrices with higher collagen density (**Figures 3B, C**). Furthermore, the nuclei of migrating CTLs in dense collagen matrices were more frequently deformed to an hour-glass shape than their counterparts in low-density collagen (**Figure 3D**). Nuclear irregularity index decreased in dense collagen (4 and 5 mg/ml) (**Figure 3E**). It is reported that in dendritic cells, the nucleus is drastically deformed when migrating through spatially restricted areas (25). Together, our results suggest that the extent of nuclear deformation is increased in dense collagen likely due to its decreased porosity.

We next examined whether nuclear deformation correlated with CTL migration in 3D. As shown in the exemplary cells, at the time points when the nucleus was deformed, the real-time velocity was high. This phenomenon was observed for all three concentrations (**Figure 3F**, **Supplementary Movies 4–6**). The respective analyses show that the migration velocity of CTLs is positively correlated to nuclear deformability as determined by the change in nuclear irregularity index (sphericity) in each density (**Figure 3G**). This observation is in good agreement with a recent report, showing that cell migration velocity positively correlated to the change in nucleus shape in 2D (28). Together, these findings suggest that deformability of the nucleus is a key factor to determine CTL migration in 3D.

### Nuclear Deformability Is Regulated by the Microtubule Network

**Figure 4:**
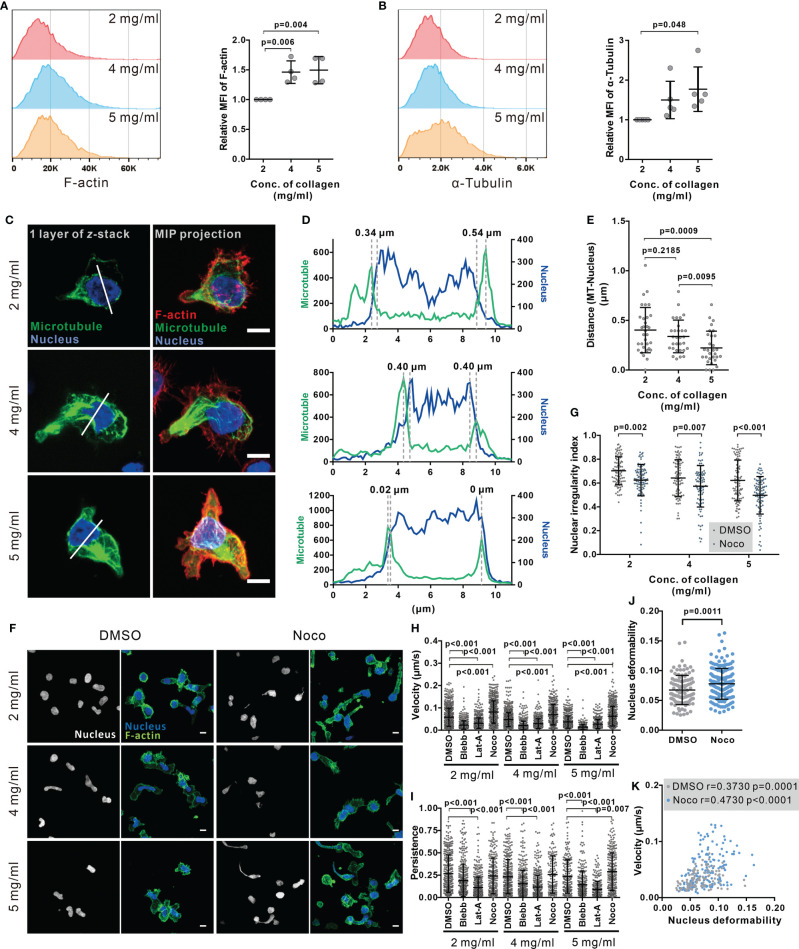
Disruption of microtubule network enhances CTL nuclear deformation and migration in dense collagen matrices. **(A, B)** Dense matrices slightly up-regulates expression of F-actin and α-tubulin. CTLs were embedded in the collagen matrix for 5 hours after polymerization and then recovered from collagen. The F-actin and α-tubulin were stained with Alexa 488-Phalloidin and anti α-Tubulin mAb antibody. The samples were analyzed with flow cytometry. Presentative donors are shown in the left panels and the quantifications are shown in the right panels. One dot represents one donor. **(C–E)** The microtubule network is located at the nucleus-surrounding region. CTLs were transfected with EMTP-3×GFP (green). 24 hours post-transfection CTLs were fixed and stained with Hoechst 33342 (blue), and Alexa 568-phalloidin (F-actin, red). Images were acquired with confocal microscopy (63× objective). MIP: maximum intensity projection. Scale bars are 5 µm. The fluorescence intensity along the random line cross nucleus depicted in **(C)** is shown in **(D)**. Distance between microtubules and nucleus is defined as the distance between two maxima as indicated in **(D)** and quantified in **(E)**. **(F**, **G)** Disruption of the microtubule network increases the level of nuclear deformation in CTLs. Collagen-embedded CTLs were treated with nocodazole (Noco, 10 µM) or DMSO for 5 hours prior to fixation. Then CTLs were stained with Hoechst 33342 (blue) and Alexa 488-phalloidin (F-actin, green). Images were acquired with confocal microscopy (63× objective). Exemplary images are shown in **(F)**. Quantification of the nuclear irregularity index in maximum intensity projection is shown in **(G)**. **(H, I)** Impact of cytoskeletal components on CTL migration in 3D. Hoechst 33342-stained CTLs were embedded in planar collagen and treated with DMSO, blebbstatin (Blebb, 50 µM), latrunculin-A (Lat-A, 50 nM), or nocodazole (Noco, 10 µM). Migration was visualized with cell observer (20× objective) at 37°C for 30 min. Migration velocity and persistence are shown in **(H, I)**, respectively. **(J, K)** Disruption of the microtubule network enhances nuclear deformability. CTLs were embedded in collagen (5 mg/ml) and then treated as in **(H, I)**. Average deformability of CTLs is shown in **(J)**. Correlation between migration velocity and nuclear deformability is shown in **(K)**. The correlation coefficient r is analyzed with Spearman’s correlation. One dot represents one cell. Scale bars are 5 µm. Results are presented as Mean ± SD from 3 donors. For statistical significance, the unpaired Student’s t-test (in **A**, **B**, **E**) or the Mann-Whitney test **(G–J)** was used.

Cytoskeleton is an essential regulator for nuclear deformation induced by mechanical forces (41, 42) as well as for cell migration (43). Therefore, we examined the contribution of key cytoskeletal components to the regulation of nuclear deformation and CTL migration in 3D. The expression of F-actin (**Figure 4A**) and microtubules (**Figure 4B**) in collagen-embedded CTLs was modestly upregulated in the dense matrices (4 mg/ml and 5 mg/ml). We analyzed the intracellular distribution of F-actin and microtubules using immuno-staining. Confocal images and live-cell imaging show that F-actin was mainly located in the CTL cortex, whereas the microtubule network was enriched around the microtubule-organizing center (MTOC) at the uropod and nucleus-surrounding areas (**Figure 4C**, **Supplementary Movie 7**) as reported by the others (44). Noticeably, the average distance of microtubule network and the nucleus was decreased upon the increase in collagen density (**Figures 4D, E**, **Supplementary Figures 6A, B**). Moreover, short distance between microtubule network and the nucleus benefits to keep the nucleus volume and sphericity in dense collagen (**Supplementary Figures 6C–G**). As it has been reported that the volume of the nucleus is limited by the microtubule network (42), we hypothesized that the microtubule network is involved in regulating CTL nuclear deformability.

To investigate this hypothesis, we used nocodazole, an inhibitor of tubulin polymerization, to abrogate the functionality of the microtubule network. We found that nuclear circularity was significantly decreased by nocodazole treatment as illustrated in the exemplary cells (**Figure 4F**) and the quantitative analysis (**Figure 4G**), indicating that with microtubules depolymerized, the nucleus is more deformable.

**Figure 5:**
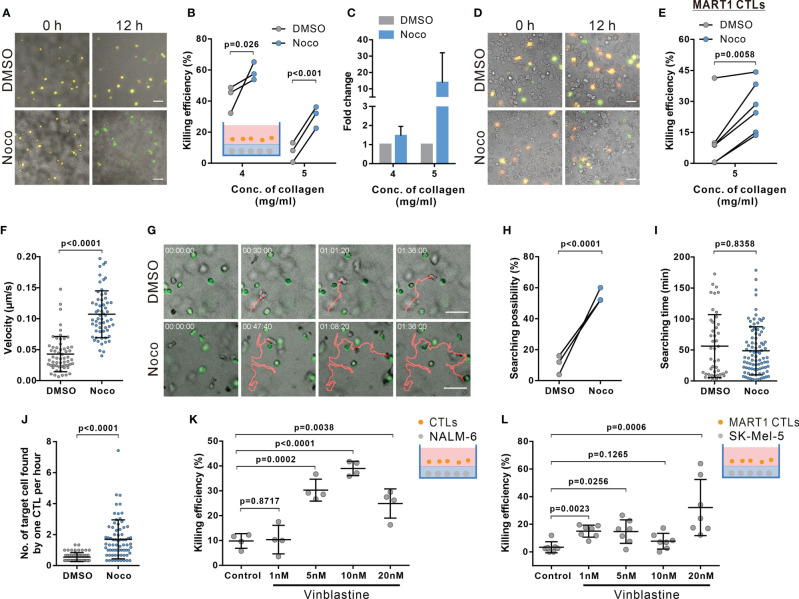
Disruption of microtubules ameliorates impaired CTL killing in dense collagen matrices. **(A–E)** Inhibition of microtubule polymerization improves CTL killing in dense collagen. Nocodazole (Noco, 10 µM) was used to disrupt the microtubule network. Images were acquired using ImageXpress (20× objective) at 0 and 12 hours. NALM-6-pCasper and bead-stimulated CTLs **(A–C)**, or SK-Mel-5 pCasper and MART1-specific CTLs **(D**, **E)** were used as target and killer cells. Representative experiments from 5 mg/ml are shown in **(A, D)**. **(F–J)** Nocodazole treatment elevates migration and searching efficiency in dense collagen. CTLs and calcein-loaded target cells (green) were embedded in planer collagen matrix (5 mg/ml) and visualized with cell observer. Migration velocity within the duration of 30 min before conjugating with target cells is shown in **(F)**. Representative migration trajectories are shown in **(G)**. The likelihood for CTLs to find target cells and the time required for CTL to find their first target cell are shown in **(H, I)**. Number of target cells found by one CTL per hour is shown in **(J)**. **(K**, **L)** Vinblastin enhances killing efficiency of CTLs in dense matrices. Respective target cells as indicated were embedded in collagen (5 mg/ml). Killing efficiency was determined at 24 hours **(K)** or 12h **(L)** after adding CTLs using ImageXpress (20× objective). The ratio between effector cells and targets is 5:1 in **(A–C, K)**. The ratio between effector cells and targets is 1:1 in **(D**, **E**, **L)**. One dot represents one donor (**B**, **H**, **K**, n = 3 donors), one independent experiment (**E**, n = 6 and **L**, n = 7 experiments), or one cell (**F**, **I**, **J**, n = 4 donors). Scale bars are 50 µm. Results are presented as Mean ± SD. Scale bars are 50 µm. For statistical significance, the paired Student’s t-test **(B**, **E**, **H)** or the Mann-Whitney test **(F**, **I–L)** were used.

Combined with the finding that nuclear deformation is correlated with CTL migration, we postulated that disruption of the microtubule network should impact CTL migration, especially in dense ECM. Analysis of migration of CTLs in collagen matrices shows that in CTLs treated with nocodazole migration velocity was enhanced at all matrix densities (**Figure 4H**); whereas persistence was only increased in collagen with 5 mg/ml (**Figure 4I**). In comparison, disruption of the actin network by latrunculin-A or abrogation of myosin IIA by blebbstatin almost abolished CTL velocity and persistency for all three collagen densities (**Figures 4H**, **5I**). Inhibition of a myosin IIA-upstream kinase Rock, or focal adhesion kinase also drastically impaired CTL velocity (**Supplementary Figure 7A**) but did not drastically change 3D migration persistence (**Supplementary Figure 7B**). Furthermore, live-cell imaging shows that the nucleus in the nocodazole-treated CTLs was more deformable than their DMSO-treated counterparts (**Figure 4J**). For CTLs, despite nocodazole-, vehicle-, or other inhibitors-treated, the migration velocity was positively correlated with the extent of nuclear deformation (**Figure 4K**, **Supplementary Figure 8**). In summary, we conclude that disruption of the microtubule network enhances CTL migration especially in dense collagen matrices, which is correlated with enhanced deformability of nucleus in CTLs.

### Disruption of Microtubules Ameliorate Impaired CTL Killing in Dense ECM

Considering the dependence of CTL migration velocity and persistence on the microtubule-network, we hypothesized that interference with microtubules should improve the impaired killing efficiency in dense ECM. Results of 3D killing assay show that indeed in dense matrix (4 mg/ml and 5 mg/ml), disruption of the microtubule network ameliorated CTL killing (**Figures 5A–C**). Moreover, to further confirm this phenomenon, we used primary human CTL clones specific for MART1 (also known as Melan-A) (31). A melanoma cell line SK-Mel-5, which endogenously, present MART1 on their surface (45), was used as target cells. The time lapse shows that in good agreement with previous results, nocodazole-treatment significantly enhanced killing efficiency of CTL clones to remove SK-Mel-5 (**Figures 5D, E**). Together, our findings suggest that the microtubule network is as a promising target to improve CTL killing against tumor cells in dense ECM.

We further confirmed that also in presence of target cells, disruption of the microtubule-network with nocodazole treatment (10 μM) promoted 3D CTL migration (**Figure 5F**), but not reduced cytotoxic protein secretion and expression (**Supplementary Figure 9**). Concomitantly, the likelihood for microtubule-disrupted CTLs showed increased velocity (**Figure 5G**) and had a higher probability to find their target cells (**Figure 5H**). Only analyzing CTL which were successful to find at least one target, nocodazole-treated CTLs needed similar times to locate their first targets compared to DMSO-treated CTLs (**Figure 5I**), there was only a slight but insignificant reduction of search time for the first target. However, on average, the number of target cells found by nocodazole-treated CTLs was significantly higher than by control CTLs (**Figure 5J**). In addition, we examined CTL viability after 12 hour-nocodazole treatment, which is the condition we used to examine killing efficiency. We found that under this condition, CTL viability in 5 mg/ml collagen was slightly reduced (**Supplementary Figure 10**). Together, we conclude that enhancement of CTL killing efficiency in dense collagen by nocodazole-treatment is due to the amelioration of CTL migration and infiltration, not due to improvement of CTL survival.

Finally, we tested vinblastine, a microtubule-inhibitor applied as a chemotherapeutic to disrupt tumor cell mitosis (46). We treated CTLs with vinblastine to determine CTL killing efficiency in dense collagen. We found that vinblastine increases both killing efficiency of primary CTLs (**Figure 5K**) and human MART1-specific CTL clones (**Figure 5L**). In summary, we conclude that disruption of the microtubule network significantly enhances CTL migration and killing efficiency in dense collagen.

## Discussion

The motility of CTLs in 3D environments, especially when moving through dense tissue matrices, is key for their search efficacy and consequent killing efficiency. In this work, we used bovine collagen matrices with three concentrations (2, 4, and 5 mg/ml) to mimic healthy tissue, soft and stiff solid tumor, mainly based on the stiffness. The storage moduli of 2 mg/ml, 4 mg/ml, and 5 mg/ml of bovine collage is 0.811 ± 0.009 kPa, 2.661 ± 0.098 kPa, and 3.640 ± 0.127 kPa, respectively (**Figure 2C**), which are in a comparable range of human healthy tissue (e.g. colon ~0.9 kPa, mammary gland ~1 kPa) and tumors (e.g. liver tumors ~2.4 kPa, breast cancer ~2.5 kPa and colon tumors ~5 kPa) (47–49). We found that two physical properties of matrices are decisive for T cell migration: pore size and stiffness (or elasticity) of the fibrils. As concentration of collagen increases, pore size gets smaller and stiffness increases, which is in line with other reports (24, 50). Our work shows that human CTLs migrate spontaneously in 3D collagen matrix. Both the speed and the persistence of their 3D migration diminish with increasing collagen concentration. The concentration-dependent correlation of porosity and stiffness can be decoupled in reconstituted collagen matrices. Keeping the concentration of collagen constant, lowering polymerization temperature increases the pore size of the fibrillar matrix, whereas pretreatment of collagen with ribose enhances the stiffness of collagen fibrils. We show that speed and persistence of human CTL migration in 3D are distinctively regulated: the speed strongly correlated with the pore size, in good agreement with previous reports (51, 52); while the persistence is mainly determined by the stiffness, in good agreement with a recent report for ovarian cancer cells (53).

For migrating human CTLs, a positive correlation between nuclear deformation and cell speed in 3D collagen matrices was observed. The nuclei of cells displayed an hourglass-like deformation in migrating CTLs, very likely through confined spaces. The diameters of the neck of hourglass (cross-section) decreases with enhanced density of collagen. The nucleus is the most rigid intracellular organelle, which provides protection of the chromatin content (54, 55). As reported in many cell types, severe deformation or even rupture of nuclei leads to DNA damage and ultimately cell death (26). In dense ECM, the enhanced nuclear deformation could therefore also lead to an elevated level of CTL apoptosis, which could eventually also contribute to dense ECM-impaired CTL killing efficiency.

Interestingly, we observed that the microtubule network is located in vicinity to the envelope of nucleus and that disruption of microtubule polymerization further enhances nuclear deformation/rupture consequently resulting in more CTL apoptosis, suggesting a protective role of the microtubule network on nuclear morphology and integrity of chromatins. Compelling evidence shows that the nuclear envelope protein lamin-A/C acts is a critical structural element required for nuclear membrane organization and stability (56). The nucleus morphology is associated with the microtubule network (**Supplementary Figure 6**) and the microtubule network could protect nucleus in coordination with lamin A/C (57). It is reported that lamin A/C is induced upon activation of T cells (58). Notably, microtubules are 300 fold more rigid than actin filaments (59). Thus, the microtubule network could provide a mechanistically stable structure surrounding the nucleus to protect the integrity of chromatins, in collaboration with lamin A/C and/or other nuclear envelope proteins.

Our live-cell imaging shows that when CTL migrate through a physical restriction, actin-driven protrusions leads the way through the confinement followed by translocation of the microtubule network along with the nucleus through the confined space. It is reported that in cells, deficiency in actomyosin-based contractility by myosin IIA depletion or inhibition of ROCK impedes contraction of the cell rear and fails to propel the nucleus through a restricted space (24, 60, 61), and profilin-1 is involved in protrusion formation in CTLs (62). The nucleus serves as a mechano-limiting factor, which determines the possibility of cells to go through physical confinement (25, 51). In our work, we found that abrogation of myosin IIA or ROCK significantly limited CTL migration in 3D collagen matrices ranging from low to intermediate-density. This is very likely due to the absence of actomyosin contraction resulting in failing translocation of nucleus through the physical restriction. Unexpectedly, disruption of microtubule polymerization with nocodazole promotes both nuclear deformation and CTL migration in low, intermediate and high density. This indicates that the microtubule network serves as an additional mechano-limiting factor in addition to the nucleus to control CTL migration in 3D, especially through restricted space (27). In comparison, in large pores/channels, the microtubule network is not the rate-limiting factors as one recent report shows that nocodazole has no effect on T cell migration through micro-channels with width equals to or beyond 6 μm (63), which already exceeds physical constrains in low-density collagen, porosity of which ranges from 2-5 μm (24). In addition, several recent studies have reported the impact of microtubule integrity on infiltration or migration of T cells *in vivo* or *ex vivo*. More specifically, inhibition of microtubule polymerization using combretastatin A-4 increases the number of infiltrating CD8^+^ T lymphocytes in tumors in a mouse model (64). Moreover, nocodazole-treatment increases mouse CTL migration velocity in tumor slices ex *vivo* (17). These results strongly suggest that microtubule disruption improves CTL migration also *in vivo*.

Integrity of both actin-cytoskeleton and the microtubule network is pivotal to execute CTL killing processes. Actin-cytoskeleton has two compensatory roles. On one hand, functionality of cortical actin is essential for TCR triggered release of lytic granules to induce destruction of target cells (65). On the other hand, recovery of cortical actin in CTLs at the contact site with the target cell terminates release of lytic granules (66). In addition, latrunculin-A treatment for target cells could also reduce target lysis-induced by perforin (67). In our work, we show that disassembly of F-actin significantly diminishes CTL motility and killing efficiency in 3D matrices. The concentration of latrunculin-A we used was 50 nM, which should only partially disassemble F-actin as CTLs could still migrate and kill under this condition. The impairment of CTL motility and the consequent reduction in searching efficiency is the primary factor for the reduced killing by disassembly of F-actin. The effect of latrunculin-A on target cells might contribute to reduced killing to some extent, but if so only as a secondary factor.

In terms of the microtubule network, re-orientation of MTOC to the IS is a hallmark for CTL activation upon recognition of target cells, which plays a key role in enriching lytic granules towards the IS (66, 68). Perturbation of the microtubule architecture in CTLs results in reduced killing efficiency but does not affect degranulation (46). In our work, although the microtubule network was disrupted by nocodazole (10 μM) to a large extent, the remaining network was sufficient to support LG release as shown in **Supplementary Figure 9A**. The role of microtubule in human CTL migration in 3D is also strongly supported by the recent study showing that perturbation of microbutules enhances migration of human CD4^+^ T cells and mouse CD8^+^ T cells in 3D (17). More importantly, the enhancement in migration of nocodazole-treated CTLs in dense collagen leads to more conjugation and a consequently elevated efficiency of target destruction. Therefore, we conclude that in dense collagen matrices, CTL motility serves as a rate-limiting factor for killing. Several microtubule inhibitors are applied as chemotherapeutic reagents, such as vinblastine and vincristine. Interestingly, we found that vinblastine indeed enhances CTL-mediated elimination of tumor cells in dense ECM. Our findings suggest that microtubule-inhibiting chemotherapeutic reagents do not only have a direct effect on abrogation of tumor cell proliferation, but also have the potential to enhance CTL killing efficiency against tumor cells in densely packed tumor microenvironment.

## Data Availability Statement

The datasets presented in this study can be found in online repositories. The names of the repository/repositories and accession number(s) can be found below: https://cloud.hiz-saarland.de/s/g3cR3BSfbs42reD.

## Author Contributions

RZ designed and performed most experiments and all the corresponding analyses if not mentioned otherwise. XZ stained collagen matrices and imaged the structure. EK carried out rheology experiments and AC helped interpret the results. WY helped with flow cytometry. KF and ES established MART1-specific T cells clones. ES established NALM-6-pCasper cell lines. DA provided expertise in pCasper-pMax and the corresponding analysis. MH helped with data interpretation and provided critical feedback on all aspects of the project. BQ and RZ generated concepts, designed experiments, and BQ wrote the manuscript. All authors contributed to the article and approved the submitted version.

## Funding

This project was funded by the Deutsche Forschungsgemeinschaft (SFB 1027; Forschungsgroßgeräte (GZ: INST 256/419-1 FUGG for the light-sheet microscope, GZ: INST 256/423-1 FUGG for the flow cytometer, and GZ: INST 256/429-1 FUGB for ImageXpress), Bundesministerium für Bildung und Forschung (BMBF, 031L0133 to MH), University of Saarland HOMFORexzellent grant (to RZ), and by the Leibniz-Gemeinschaft (INM Fellow to BQ).

## Conflict of Interest

The authors declare that the research was conducted in the absence of any commercial or financial relationships that could be construed as a potential conflict of interest.

## Publisher’s Note

All claims expressed in this article are solely those of the authors and do not necessarily represent those of their affiliated organizations, or those of the publisher, the editors and the reviewers. Any product that may be evaluated in this article, or claim that may be made by its manufacturer, is not guaranteed or endorsed by the publisher.
