# Supplementary material for: Targeting the Microtubule-Network Rescues CTL Killing Efficiency in Dense 3D Matrices
Source: Front Immunol. 2021 Aug 17;12:729820. doi: 10.3389/fimmu.2021.729820 (PMC8416057; doi:10.3389/fimmu.2021.729820)
Supplement: Supplementary file 1 [file DataSheet_1.pdf]

# *Supplementary Material*

## Supplementary Figures

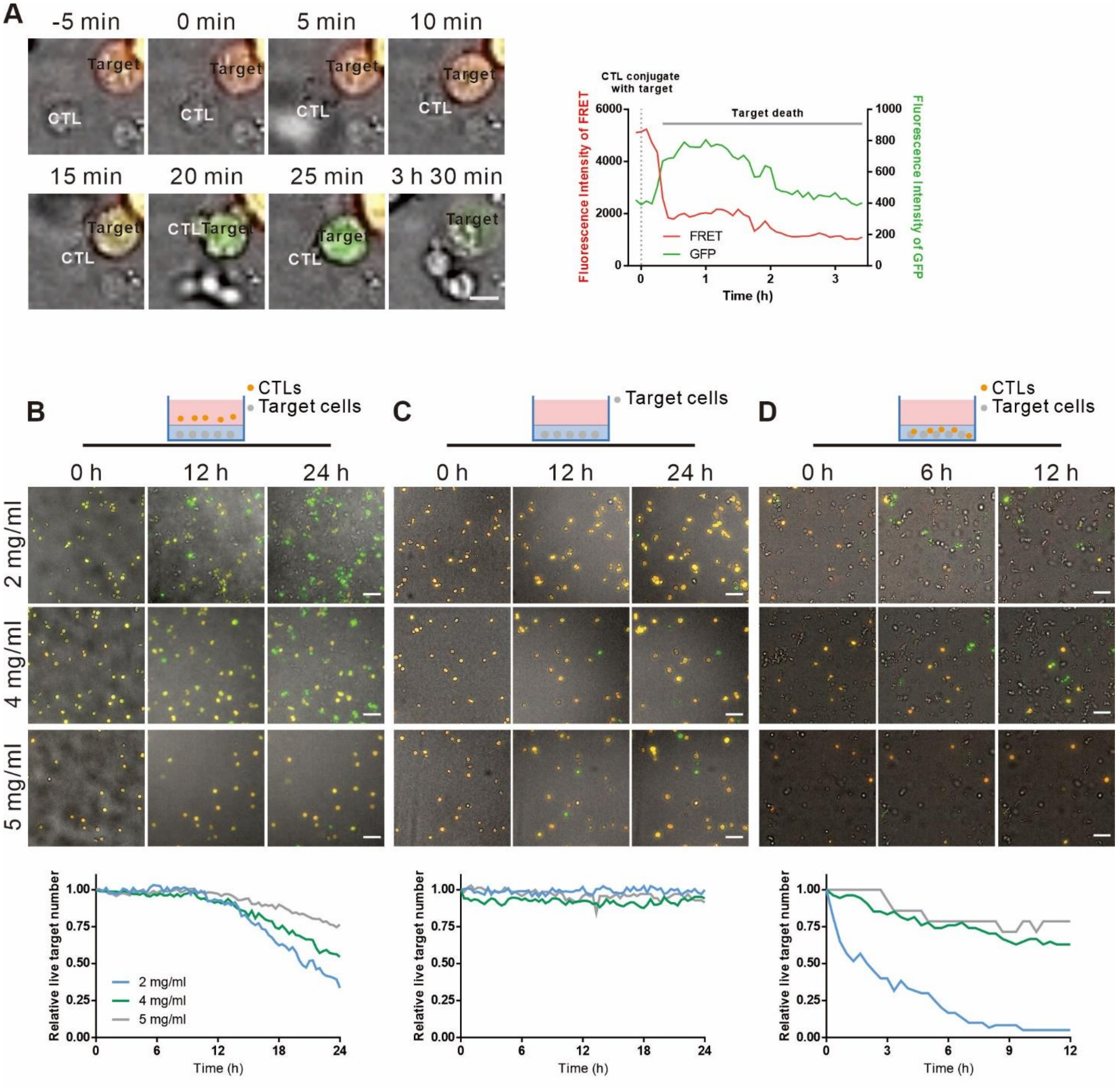


**Supplementary Figure 1. Characterization of target lysis in 3D by CTLs. A** Determination of apoptosis versus necrosis using pCasper-expressing target cells. Target cells (SEA/SEB pulsed NALM-6-pCasper) were embedded in collagen with CTLs. Images were acquired using ImageXpress at 37ºC with 5% $CO_2$ every 5 min for 12 hours. The time 0 was determined when CTL contacts with

the target cell. Target cells in yellow-orange were alive as indicated by FRET signal. When the target cell was undergoing apoptosis, the fluorescence was switched to green. In necrotic target cells, all the fluorescence would go down abruptly. The dynamics of fluorescence were shown in the right panel. The scale bar is 10 µm. Results are from 3 donors. **B-D** Killing kinetics in 3D. Target cells (SEA/SEB pulsed NAML-6-pCasper) were embedded in collagen in absence (**B**, **C**, Target cell only) or presence of CTLs (**D**, Target cell + CTL) in half-area 96-well plates. In **B**, CTLs were added into the wells after polymerization of collagen. Images were acquired using ImageXpress every 20 min for 24 h (**B**, **C**) or 12 h (**D**). Time lapse images and the corresponding killing kinetics of one representative donor are shown in the upper and lower panels, respectively. Scale bars are 50 µm. Results are from 3 donors (in **B**, **C**) or 5 donors (in **D**).

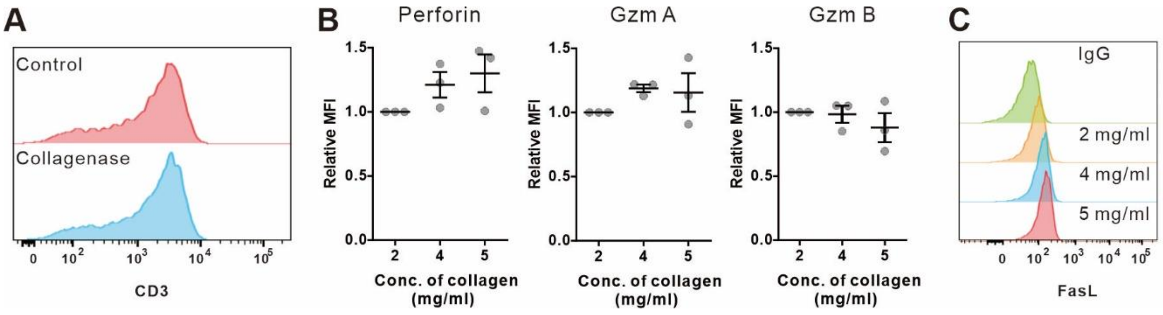


**Supplementary Figure 2. Impact of collagen density on lytic granule pathway. A** Collagenase I does not degrade surface protein on T cells. Jurkat T-cells were treated with or without collagenase for 7 min at 37$^o$C, followed by fixation and CD3 staining without permeabilization. The samples were analyzed with flow cytometry. **B**, **C** CTLs were embedded in collagen matrices with indicated densities and were kept at 37°C with 5% $CO_2$ for 5 h. Then collagen matrices were digested by collagenase I for 7 min at 37$^o$C. **B** Expression of perforin, granzyme A (GzmA), or granzyme B (GzmB) was stained with respective antibodies in fixed and permeabilized CTLs, and measured with flow cytometry (from 3 donors). **C** FasL expression was stained FasL antibody in fixed and permeabilized CTLs, and determined with flow cytometry (from 2 donors). In **B**, one dot represents one donor. Results are presented as Mean±SD.

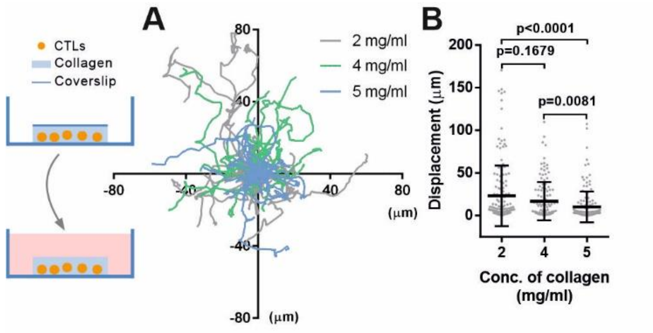


**Supplementary Figure 3. CTL migration is impaired in dense ECM.** CTLs were embedded in planar collagen. **A** The trajectories of CTLs migration in planar collagen with different concentrations. **B** The displacement of CTL migration. Migration was visualized at 37°C for 30 min with Cell Observer

(20× objective). One dot stands for one cell. All cells are from 3 donors. Results are presented as Mean±SD. P values were accessed with the Mann-Whitney test.

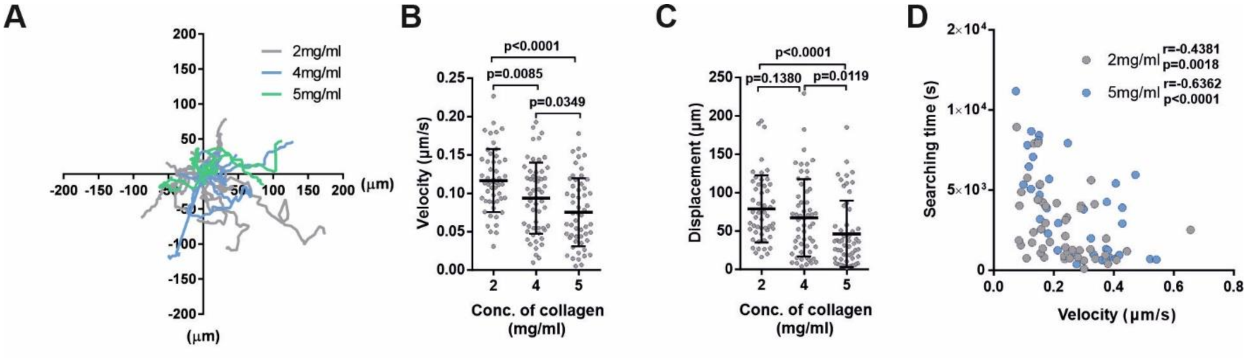


**Supplementary Figure 4. CTL motility is significantly sabotaged in dense ECM with target cells present.** CTLs were embedded in planar collagen matrices with indicated densities in presence of target cells (SEA/SEB-pulsed Raji cells) and were visualized at 37°C every 30 sec for 3 hours. CTLs were tracked manually by Fiji with tracking plugin. The trajectories (**A**), velocity (**B**), and displacement (**C**) of CTLs migration, were from a duration of 30 min before they got in contact with a target cell. **D** CTL migration velocity is negatively correlated to the time of CTL searching the first target cell. One dot or on trajectory stands for one cell. Results are from 3 donors. Results are presented as Mean±SD. P values were accessed with unpaired Student's t-test (in **B**) or the Mann-Whitney test (in **C**). The correlation coefficient r is analyzed with Spearman's correlation in **D**.

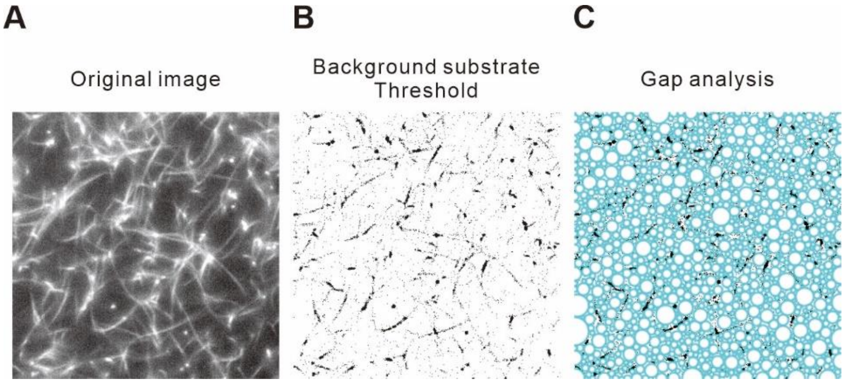


**Supplementary Figure 5. Structure of type I collagen. A** Visualization of the collagen structure of Type I collagen (2 mg/ml) from bovine was stained with Atto 488 NHS ester, and visualized using light-sheet microscopy with a 20× objective. As elaborated in Methods, the background was subtracted (**B**), and the pores were identified by the Fiji ImageJ (BIOP version) with Max Inscribed Circles plugin (**C**), from which diameters of pores were quantified.

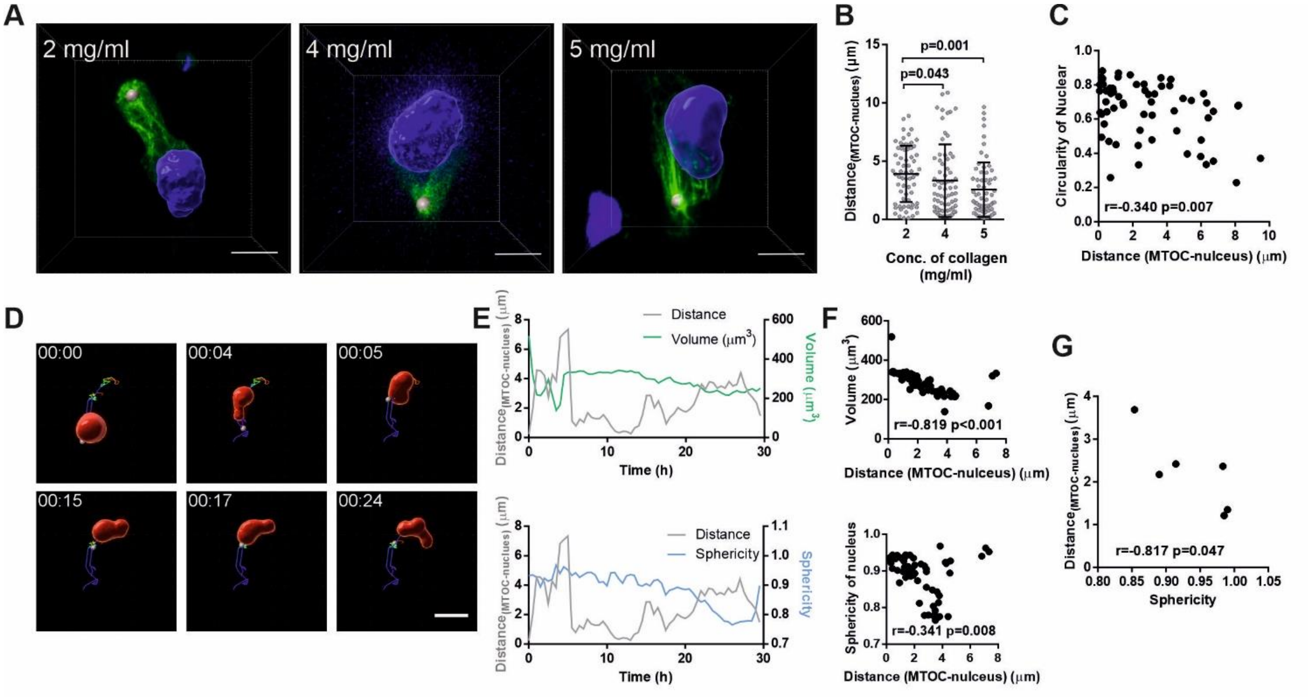


**Supplementary Figure 6. Microtubule network close to the nucleus to protect the nucleus shape and volume in dense collagen. A**, **B** The distance between MTOC and the nucleus is smaller in dense collagen. **A** Microtubule network was labeled with EMTP-3×GFP in Green. The nucleus was labeled with Hoechst 33342 in blue. MTOC (white spots) and nucleus (blue surface) were detected with Imaris 9.6. Scale bars are 10 µm. **B** The distance between MTOC and the nucleus was analyzed with Imaris 9.6. **C** The shorter distance between MTOC and nucleus keeps nucleus higher circularity of nucleus in 2D projection after CTLs were embedded in 5 mg/ml collagen. The correlation coefficient r is analyzed with Spearman's correlation. One dot stands for one cell. Data are from 3 independent experiments. **D-G** In 5 mg/ml collagen, The shorter distance between MTOC and nucleus keeps the nucleus higher sphericity and volume of the nucleus during migration. The nucleus and microtubule network was labeled with pmCherry-C1 mCherry-NLS and pmEGFP_a_tubulin_C1, respectively. **D** MTOC (white spots) and nucleus (red surface) were detected with Imaris 9.6. The distance between MTOC and the nucleus was analyzed with Imaris 9.6, as well. Scale bars are 5 µm. **E** The dynamic of the volume and sphericity of the nucleus and distance between MTOC and the nucleus. **F** The distance between MTOC and the nucleus was negatively correlated to the volume and sphericity of the nucleus during migration. One dot stands for one time point from **E**. The correlation coefficient r is analyzed with Spearman's correlation. **G** The average distance between MTOC and the nucleus was negatively correlated to the average sphericity of the nucleus in different cells. The correlation coefficient r is analyzed with Pearson's correlation. The data of **D-G** are from one experiment.

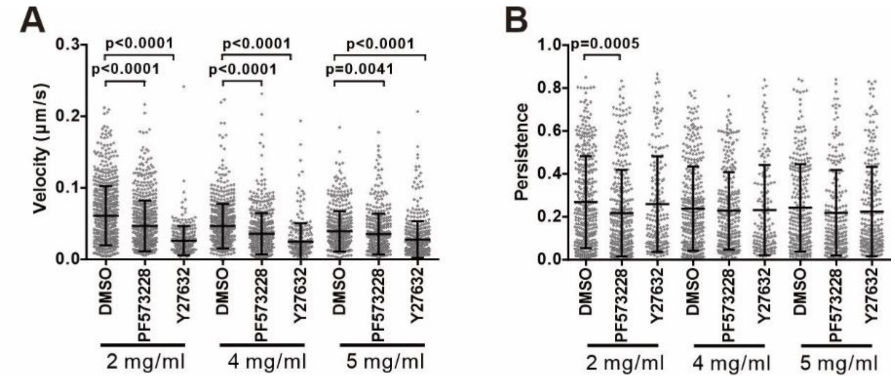


**Supplementary Figure 7. FAK and ROCK are essential for CTL migration in 3D.** CTLs were embedded in planar collagen matrices with indicated densities in the presence of the correspondent inhibitor or DMSO and were visualized at 37°C with 5% $CO_2$ for 30 min. CTLs were tracked automatically by Imaris 8.1.2. FAK inhibitor PF573228 (20 µM) and ROCK inhibitor Y27632 (10 µM) were applied. One dot represents one cell. Results are presented as Mean±SD. The results of Y27632 are pooled from 2 donors. Other results are pooled from 3 donors. The p values were accessed with the Mann-Whitney test.

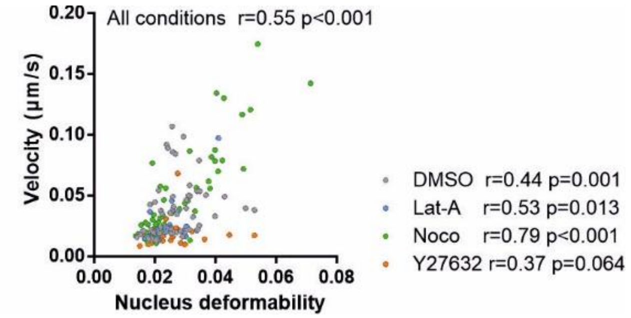


**Supplementary Figure 8. In dense collagen, the nucleus deformability is highly positively correlated to migration velocity.** CTLs migration and nucleus deformation was measured in planar 5 mg/ml collagen matrices. ROCK inhibitor Y27632 (10 µM), F-actin polymerization inhibitor latrunculin-A (50 nM), or microtubule polymerization inhibitor nocodazole (10 µM) were applied. One dot stands for one cell. The data of Y27632 are from 1 donor, others are from 2 donors. The correlation coefficient r is analyzed with Spearman's correlation.

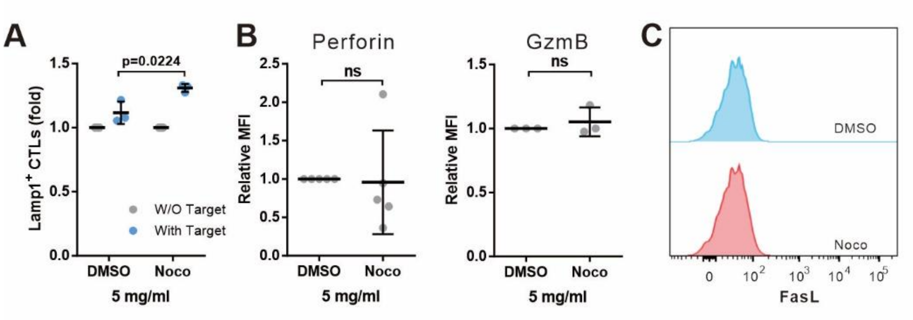


**Supplementary Figure 9. Impact of microtuble-network disruption on the expression of cytotoxic proteins**. CTLs were embedded in collagen matrices with indicated densities presence (**A**) or in absence (**B**, **C**) of target cells and were kept at 37°C with 5% $CO_2$ for 4 h (**A**) or 5 h (**B**, **C**). **A** Nocodazole-treatment enhanced relative ratio of lamp$^+$ CTLs. Nocodazole treatment does not affect the expression perforin, granzyme B (GzmB) (**B**), and FasL (**C**) significantly. The protein level was measured with the flow cytometer. Results are presented as Mean±SD. One dot stands for one donor. The data are from 3 donors, except **B** (left panel), which are from 5 donors. P values were accessed with the unpaired Student's t-test.

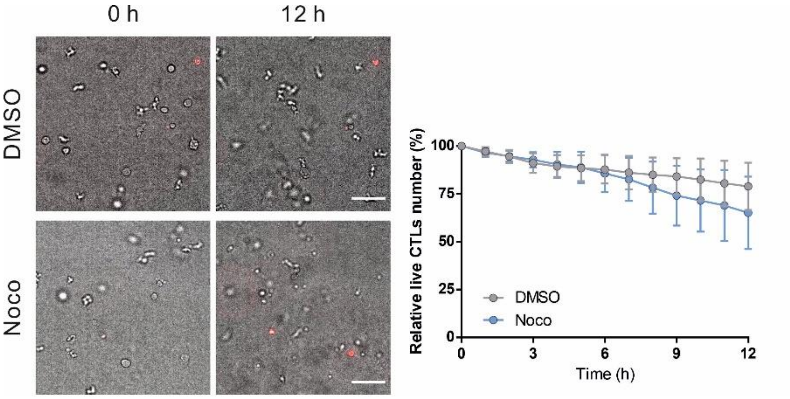


**Supplementary Figure 10. Impact of microtuble disruption on CTL viability.** Viability of CTLs was determined by propidium iodide in presence in the medium. CTLs were embedded in 5 mg/ml collagen matrices with medium containing either DMSO or nocodazole (10 µM). Images were acquired at 37ºC with 5% $CO_2$ every 1 h for 12 h. Results are presented as Mean±SD. The data are from 3 donors. Scale bars are 100 µm.

## Supplementary Movies

**Movie 1. CTL migration trajectories measured by lightsheet microscopy in 2 mg/ml collagen matrices.** Nuclei were labeled with overexpression of Histone 2B-GFP. Migration was visualized using light-sheet microscopy (20× objective) at 37°C for 30 min with an interval of 30 sec. Nuclei movement was tracked with spot tacking function of Imaris 8.1.2. Scale bar is 40 µm.

**Movie 2. CTL migration trajectories measured by lightsheet microscopy in 4 mg/ml collagen matrices.** Nuclei were labeled with overexpression of Histone 2B-GFP. Migration was visualized using light-sheet microscopy (20× objective) at 37°C for 30 min with an interval of 30 sec. Nuclei movement was tracked with spot tacking function of Imaris 8.1.2. Scale bar is 40 µm.

**Movie 3. CTL migration trajectories measured by lightsheet microscopy in 5 mg/ml collagen matrices.** Nuclei were labeled with overexpression of Histone 2B-GFP. Migration was visualized using light-sheet microscopy (20× objective) at 37°C for 30 min with an interval of 30 sec. Nuclei movement was tracked with spot tacking function of Imaris 8.1.2. Scale bar is 40 µm.

**Movie 4. Nuclei shape dynamics of CTLs during migration in 2 mg/ml collagen.** CTLs transfected with Histone 2B-GFP (to label nuclei, green) and LifeAct-mRuby (to label actin, red) were embedded in collagen. Migration was visualized using light-sheet microscopy (20× objective) at 37°C for 30 min with an interval of 30 sec. Nuclei dynamics were tracked with surface tacking function of Imaris 8.1.2. The trajectory was performed with speed statistic shown in raibow colour (0.01-0.3 µm/s). Scale bar is 10 µm.

**Movie 5. Nuclei shape dynamics of CTLs during migration in 4 mg/ml collagen.** CTLs transfected with Histone 2B-GFP (to label nuclei, green) and LifeAct-mRuby (to label actin, red) were embedded in collagen. Migration was visualized using light-sheet microscopy (20× objective) at 37°C for 30 min with an interval of 30 sec. Nuclei dynamics were tracked with surface tacking function of Imaris 8.1.2. The trajectory was performed with speed statistic shown in raibow colour ( 0.01-0.3 µm/s). Scale bar is 10 µm.

**Movie 6. Nuclei shape dynamics of CTLs during migration in 5 mg/ml collagen.** CTLs transfected with Histone 2B-GFP (to label nuclei, green) and LifeAct-mRuby (to label actin, red) were embedded in collagen. Migration was visualized using light-sheet microscopy (20× objective) at 37°C for 30 min with an interval of 30 sec. Nuclei dynamics were tracked with surface tacking function of Imaris 8.1.2. The trajectory was performed with speed statistic shown in raibow colour ( 0.01-0.3 µm/s). Scale bar is 10 µm.

**Movie 7. Microtubule network and F-actin dynamic during CTL migration in 4 mg/ml collagen matrices.** CTLs were transfected with EMTP-3×GFP (to label microtubules, green) and LifeAct-mRuby (to label acin, red). Migration was visualized using light-sheet microscopy (20× objective) at 37°C for 30 min with an interval of 6 sec. Scale bar is 7 µm.
